# Epigenetic Transgenerational Inheritance of Altered Sperm Histone Retention Sites

**DOI:** 10.1038/s41598-018-23612-y

**Published:** 2018-03-28

**Authors:** Millissia Ben Maamar, Ingrid Sadler-Riggleman, Daniel Beck, Michael K. Skinner

**Affiliations:** Center for Reproductive Biology, School of Biological Sciences, Washington State University, Pullman, WA 99164–4236 USA

## Abstract

A variety of environmental toxicants and factors have been shown to induce the epigenetic transgenerational inheritance of disease and phenotypic variation. Epigenetic alterations in the germline (sperm or egg) are required to transmit transgenerational phenotypes. The current study was designed to investigate the potential role of histones in sperm to help mediate the epigenetic transgenerational inheritance. The agricultural fungicide vinclozolin and the pesticide DDT (dichlorodiphenyltrichloroethane) were independently used to promote the epigenetic transgenerational inheritance of disease. Purified cauda epididymal sperm were collected from the transgenerational F3 generation control and exposure lineage male rats for histone analysis. A reproducible core of histone H3 retention sites was observed using an H3 chromatin immunoprecipitation (ChIP-Seq) analysis in control lineage sperm. Interestingly, the same core group of H3 retention sites plus additional differential histone retention sites (DHRs) were observed in the F3 generation exposure lineage sperm. Although new histone H3 retention sites were observed, negligible change in histone modification (methylation of H3K27me3) was observed between the control and exposure lineages. Observations demonstrate that in addition to alterations in sperm DNA methylation and ncRNA previously identified, the induction of differential histone retention sites (DHRs) also appear to be involved in environmentally induced epigenetic transgenerational inheritance.

## Introduction

A large number of environmental factors have been shown to promote the epigenetic transgenerational inheritance of disease and phenotypic variation^1^. These include toxicants such as the agricultural fungicide vinclozolin^2^ and pesticide DDT (dichlorodiphenyltrichloroethane), as well as nutritional caloric restriction or high fat diets, and stress^3^. This epigenetic transgenerational inheritance phenomenon is highly conserved and appears in a wide variety of species including plants, insects, fish, birds and mammals such as humans^1^. This non-genetic form of inheritance requires epigenetic modifications of the germline (sperm and egg) to transmit an altered epigenome to the early embryo which can impact the epigenetics and transcriptomes of all subsequently derived somatic cells^1,2^. These ancestral exposures have been shown to promote a variety of different transgenerational diseases, abnormal pathologies and phenotypic variations^1^, in some situations for hundreds of generations^1,4^. Previous literature suggests environmentally induced epigenetic transgenerational inheritance will be a component of disease etiology and contribute to the developmental basis of health and disease.

The initial epigenetic alteration in the transgenerational germline (sperm) was found to be differential DNA methylation^2,5^ and has now been observed in a variety of species^6^. Subsequently alterations in non-coding RNA (ncRNA) were found to be present in the transgenerational sperm^7,8^ and injection of sperm ncRNA into the egg was found to promote similar transgenerational phenotypes^8^. In worms or insect species such as *C*. *elegans* and *D*. *melanogaster* (drosophila) that have low levels of DNA methylation, the transgenerational phenotypes observed were found to be associated with altered histone modifications^9^. In *C*. *elegans* and drosophila transgenerational phenotypes appearing in response to abnormal nutrition or heat were found to be transmitted for 10–50 generations and involved histone modifications (methylation or acetylation)^9^. The potential role of histones in environmentally induced epigenetic transgenerational inheritance of other organisms such as mammals, humans, plants and fish has not been previously reported^1^.

A significant amount of histone research has been focused on histone modifications (methylation or acetylation) associated with somatic cell gene expression^10–14^. Interestingly, the majority of histones are removed during spermatogenesis and replaced with protamines to allow the compaction of DNA into the head of the sperm^15^. One of the predominant histone modifications on retained histones in sperm observed is the H3K27me3 repressive mark. Only 5–15% of the mammalian genome retains histones in sperm, depending on the species. Several laboratories have demonstrated the presence of conserved histone retention sites in sperm that are proposed to influence early embryonic development^16–18^. Critical developmental genes can be associated with these retained histone sites from mouse to human sperm^16,19^. The possibility that histone retention sites may be altered during environmentally induced epigenetic transgenerational inheritance has not been reported. The current study used vinclozolin or DDT to induce the epigenetic transgenerational inheritance of disease in rats to investigate the potential that histone modifications or retention sites may be affected in the transgenerational rat sperm.

## Results

The experimental design involved gestating female rats (F0 generation) being exposed transiently to vehicle (DMSO, dimethylsulfoxide) treatment for control, or vinclozolin or DDT exposure during days 8–14 of fetal development. The offspring (F1 generation) were aged and bred (without any sibling or cousin breeding) to generate the F2 generation that were then bred to generate the F3 generation control lineage, vinclozolin lineage or DDT lineage. The F3 generation males were aged to postnatal day 120 (P120) for cauda epididymal sperm collection. The epididymal sperm were collected and briefly sonicated to eliminate any somatic cell contamination and partially remove sperm tails. Chromatin was isolated from purified cauda epididymal sperm and fragmented (200–250 bp) then immunoprecipitated with anti-histone H3 antibody or anti-H3K27me3 antibody. The DNA from the chromatin immunoprecipitation (ChIP) was processed and sequenced for histone ChIP-Seq analysis. The bioinformatics analysis identified the histone retention sites and potential differences between exposures.

**Figure 1:**
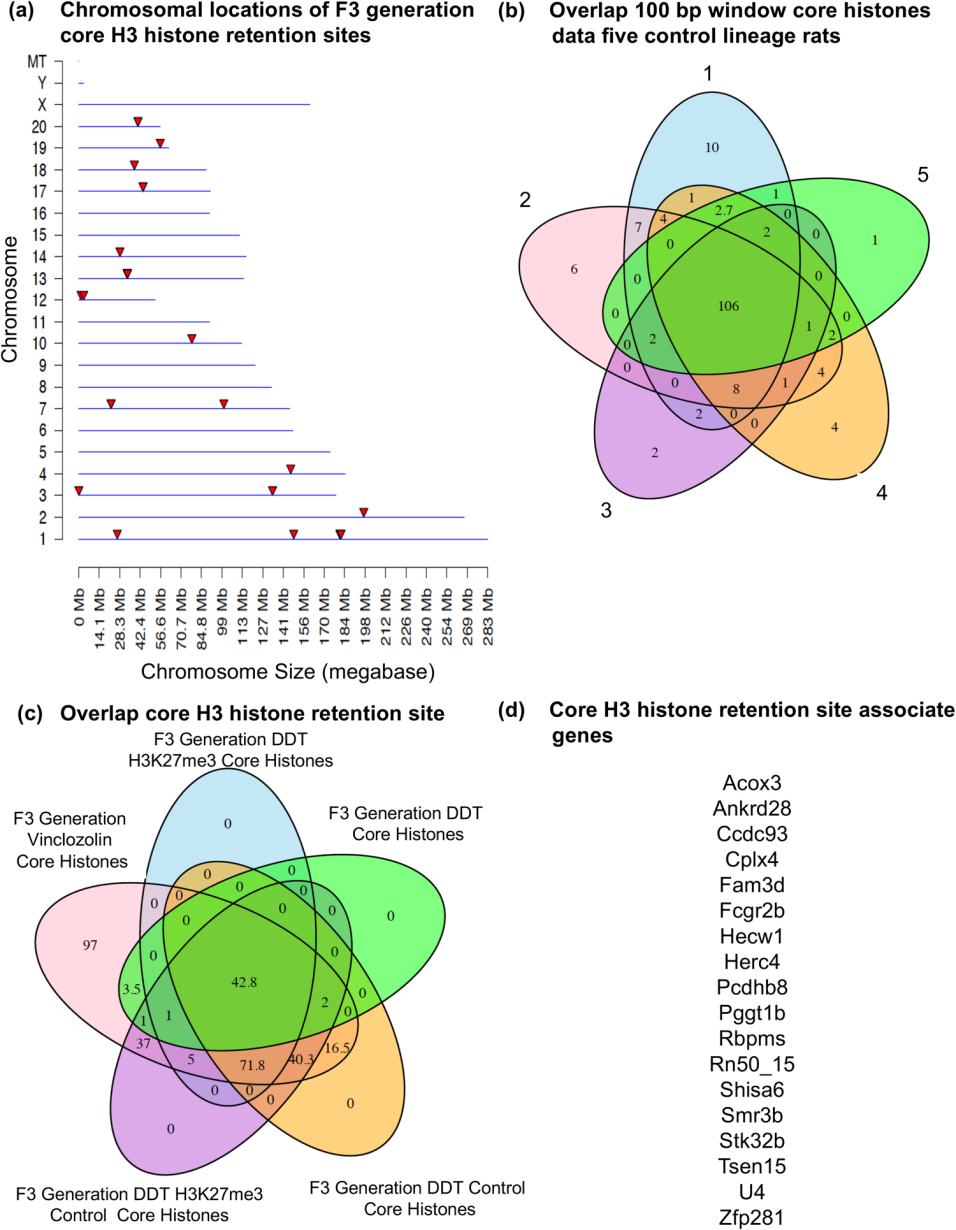
Sperm core histone retention sites. (**a**) Chromosomal locations of the core histone H3 retention sites in sperm identified with red arrowhead on different chromosomes. Only sites that overlap in all samples are shown. (**b**) Comparison of sperm core histone retention sites between five different control lineage rats. The Venn diagram shows the overlap between sperm from five different rats for significant 100 bp windows of histone retention sites. The sites are defined using a read depth threshold of 150. (**c**) Overlap of core histone retention sites between the F3 generation DDT H3K27me3, DDT, vinclozolin and associated control core histones. (**d**) List of core histone site associated genes.

The analysis of histone retention sites in the control and exposure lineage F3 generation sperm identified a highly reproducible and conserved core of H3 histone retention sites using the H3 histone ChIP-Seq analysis, Fig. 1a. These histone retention sites were on a number of different chromosomes and were predominantly 1 kb in size with some being as large as 10 kb in length. Analysis of the biological variation in the histone retention sites within the control lineage using sperm from five different individual males demonstrated that the vast majority (90%) of histone retention sites were common across animals, with less than 10% of the sites variable among the five animals, Fig. 1b. In the event the read depth threshold was reduced from 150 reads used to 25 reads, the level of variation between animals increased with approximately 50% of the histone retention sites common across animals. Therefore, this independent trial with five different individual rat sperm demonstrates a highly reproducible core set of histone sites. Interestingly, the core histone retention sites in the F3 generation control lineage were present and generally not altered in either the DDT or vinclozolin F3 generation lineage sperm, Fig. 1c.

**Figure 2:**
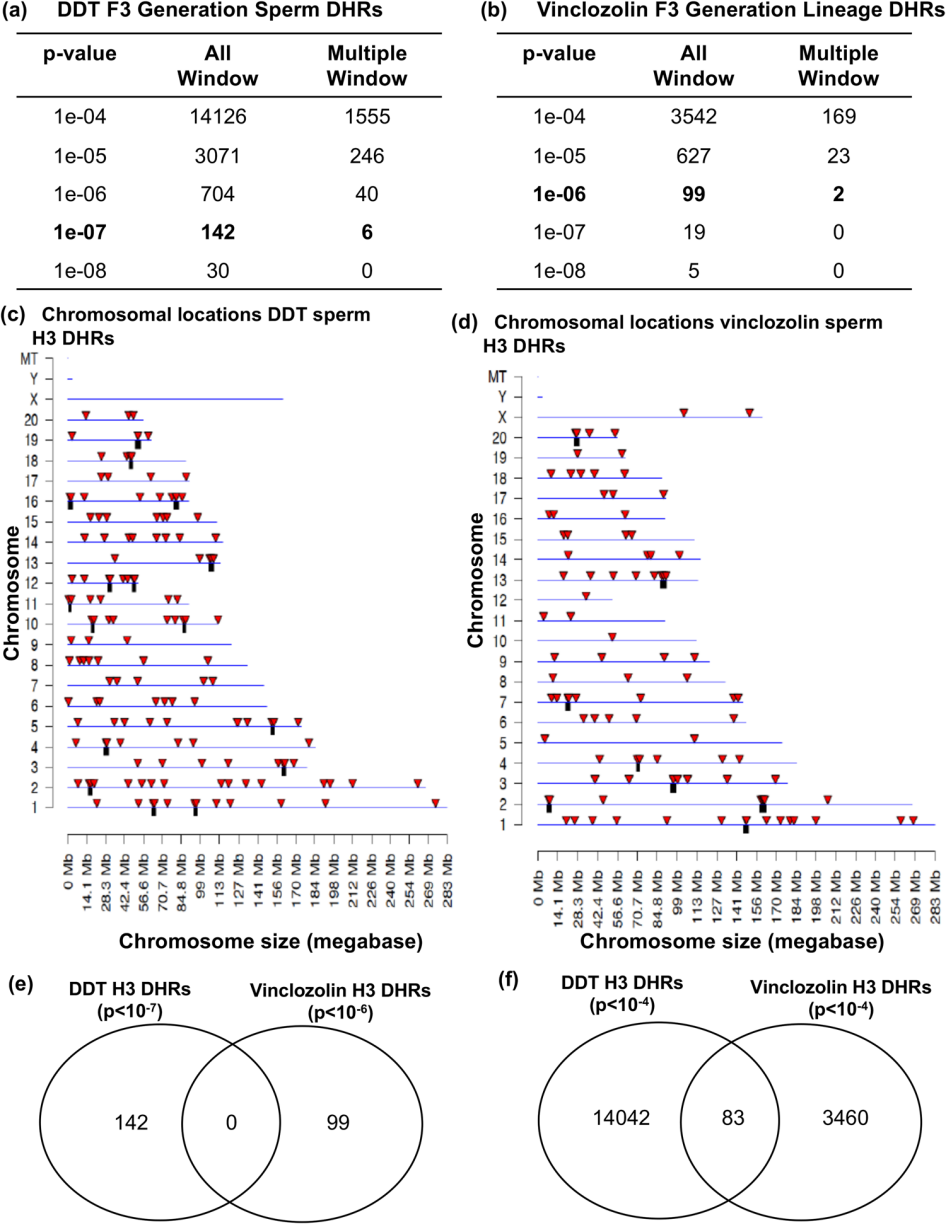
Sperm differential histone retention sites (DHRs). (**a**) DDT F3 generation sperm DHRs and (**b**) vinclozolin F3 generation lineage DHRs. The number of DHRs found using different p-value cutoff thresholds. The all window column shows all DHRs. The multiple window column shows the number of DHRs containing at least two significant windows. (**c**) DDT sperm DHR locations on the individual chromosomes. All DHRs at a p-value threshold of 1e-07 are shown. (**d**) Vinclozolin sperm DHR locations on the individual chromosomes. All DHRs at a p-value threshold of 1e-06 are shown. (**e**) Overlapping DMRs between the generation sperm DHRs F3 using p < 1e-06 for the vinclozolin results and p < 1e-07 for the DDT. (**f**) Overlapping DHRs between the two F3 generation DHRs using p < 1e-04 for the vinclozolin and p < 1e-04 for the DDT.

**Figure 3:**
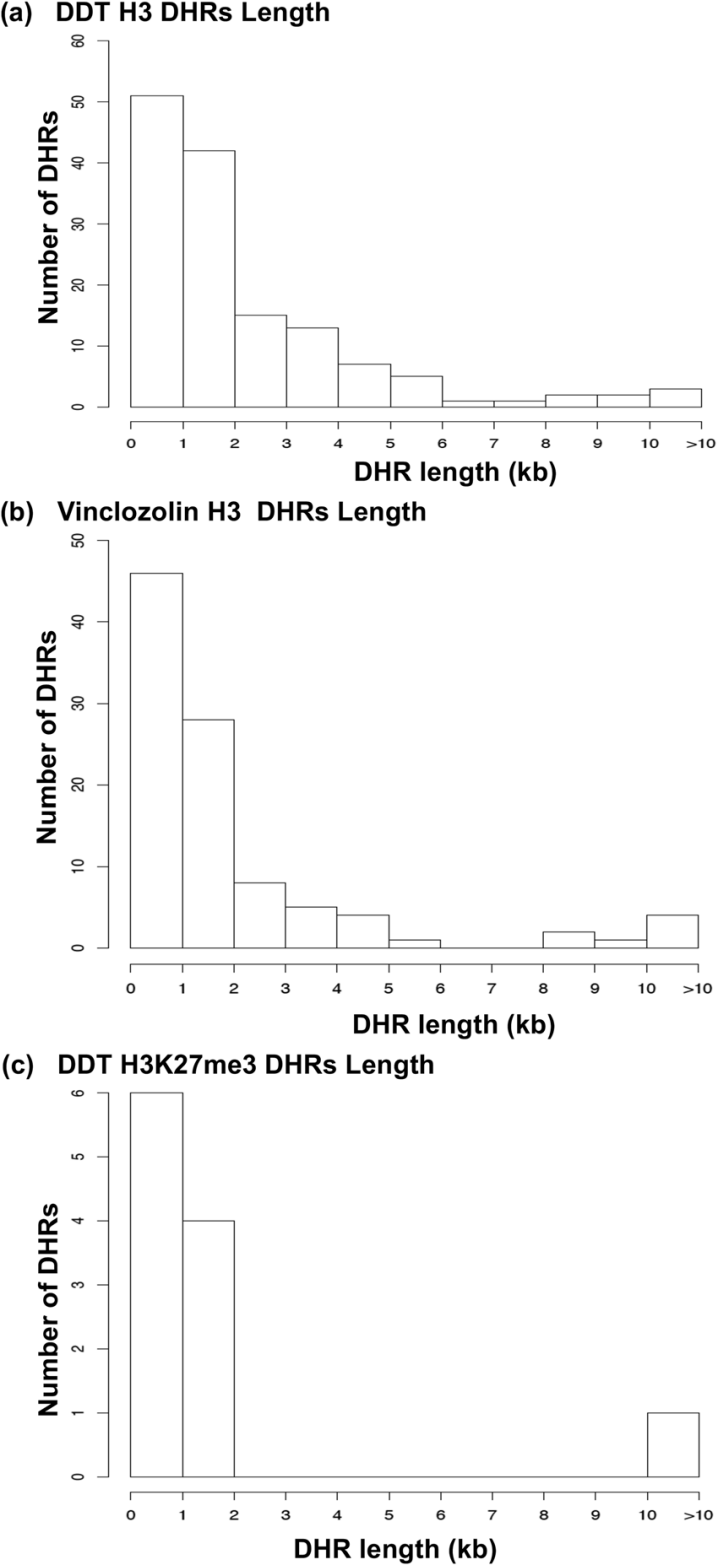
DHRs lengths. (**a**) The DDT F3 generation sperm DHR lengths (kilobases). All DHRs at a p-value threshold of 1e-07 are shown. (**b**) The vinclozolin F3 generation sperm DHRs lengths (kilobases). All DHRs at a p-value threshold of 1e-06 are shown. (**c**) The DDT H3K27me3 DHRs lengths (kilobases). All DHRs at a p-value threshold of 1e-06 are shown.

Analysis of histone retention in transgenerational F3 generation control versus vinclozolin or DDT lineage sperm identified differential histone retention sites (DHRs). Bioinformatics was used to identify statistically significant read depth differences between the control versus vinclozolin or control versus DDT lineage ChIP-Seq analysis. The number of transgenerationally altered H3 histone retention sites for the DDT lineage sperm (Fig. 2a) and vinclozolin lineage sperm (Fig. 2b) showed significant differences from the control. Since the core histone retention sites did not change (Fig. 1c), they are generally not present as DHRs, Fig. 2. The additional H3 DHRs at several different p-value thresholds are presented for all sites and those sites with multiple (≥2) adjacent significant 100 bp windows are presented. The chromosomal locations for the DDT (p < 10^−7^) H3 DHRs are presented in Fig. 2c and for vinclozolin (p < 10^−6^) in Fig. 2d. The H3 DHRs are indicated with arrowheads and clusters of DHRs with black boxes. The size of the DHRs is predominantly 1–5 kb in size with some being greater than 10 kb, Fig. 3. Therefore, both vinclozolin and DDT promoted the epigenetic transgenerational inheritance of differential H3 histone retention sites compared to the control lineage core histone retention sites. Interestingly, the H3 DHRs identified for the vinclozolin versus DDT transgenerational sperm had negligible overlap, Fig. 2f. Exposure specific DHRs were observed. However, as mentioned above, the core H3 histone retention sites in the control lineage (Fig. 1a and c) are present and not altered.

**Figure 4:**
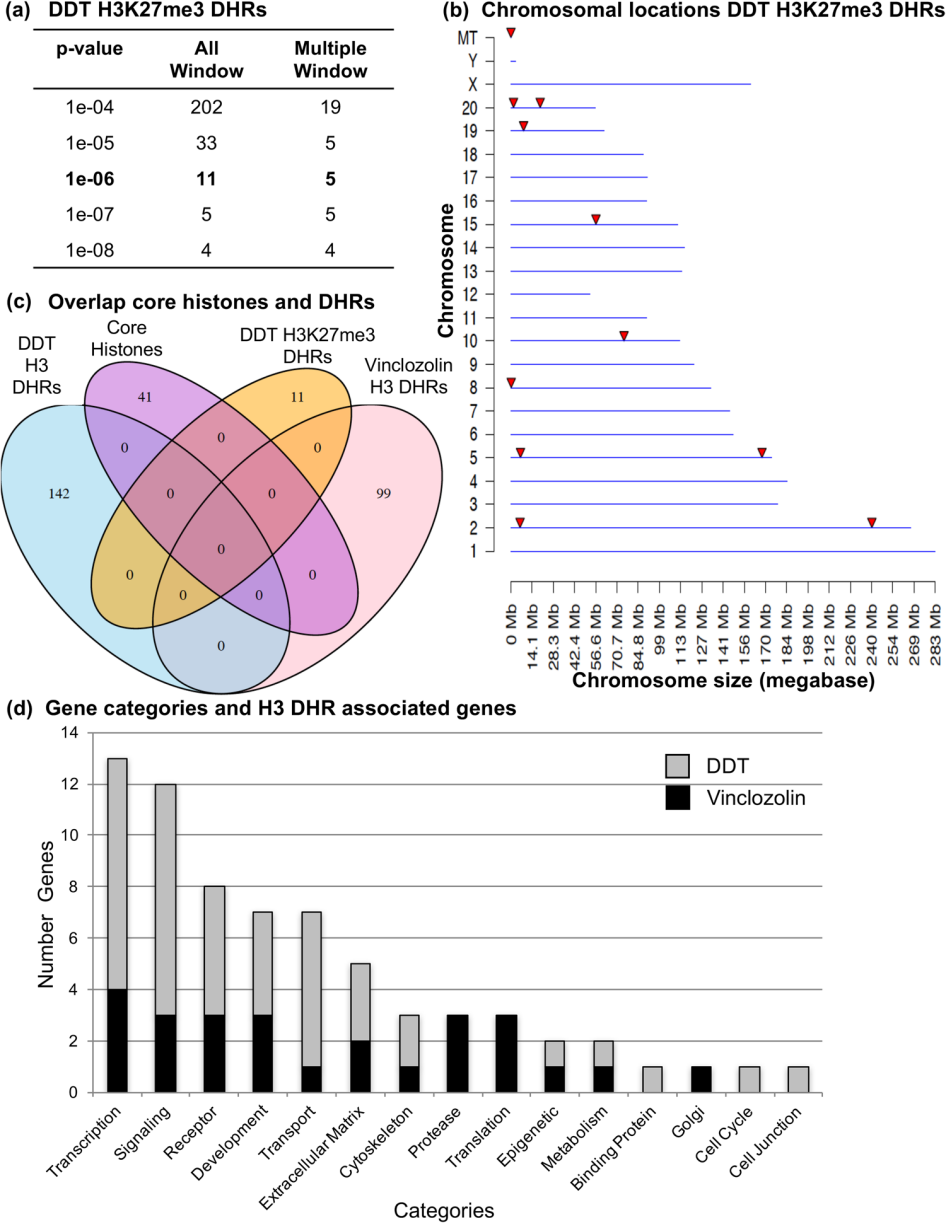
DDT sperm H3K27me3 DHRs. (**a**) The number of H3K27me3DHRs found using different p-value cutoff thresholds. The all window column shows all DHRs. The multiple window column shows the number of DHRs containing at least two significant windows. (**b**) Chromosomal DDT H3K27me3 DHRs locations on the individual chromosomes. All DHRs indicated with arrowheads at a p-value threshold of 1e-06 are shown. (**c**) Overlap of the core histones and the DDT H327me3 DHRs and the DDT or vinclozolin F3 generation sperm DHRs. (**d**) DHR associated gene categories and DHR associated genes for DDT DHRs (gray bar) and vinclozolin DHRs (black bar) with the number of genes versus the specific gene categories presented.

The majority of histone studies in sperm have focused on histone modifications and not histone retention^10–14^. Therefore, in addition to the H3 histone ChIP-Seq analysis, histone methylation was also assessed. The primary histone modification present and studied in sperm is the repressive H3K27me3 methylation^20,21^. Using the control and DDT lineage F3 generation sperm a ChIP-Seq analysis was performed to determine potential changes in H3K27 methylation. For this analysis we only used the control versus DDT lineage comparison and not vinclozolin exposure. This was due to the robust DDT differences observed and lack of sufficient material for the vinclozolin lineage. A minimal number of H3K27me3 DHRs had associated methylation alterations observed in a comparison of control versus DDT F3 generation sperm, Fig. 4a. The chromosomal locations of the H3K27me3 DHRs at p < 10^−6^ are presented in Fig. 4b. Analysis of these H3K27me3 DHRs with the DDT induced transgenerational H3 DHRs (Fig. 4c) demonstrated no overlap. Therefore, the DDT induced transgenerational H3 DHRs are unique from the DDT induced H3K27me3 alterations. Histone H3 retention alterations appear more predominant than histone H3K27me3 modification alterations during the epigenetic transgenerational inheritance phenomenon.

The genes associated (within 10 kb of gene) with the control core H3 histone retention sites shown in Fig. 1a are listed in Fig. 1d. These genes and functions were compared to recently reported histone retention sites in sperm^10,18,22,23^. Only one gene “T” or “BRA” was found to be in common and previously proposed to be important in early embryo development^24^. The majority of the core histone sites were reproducible (Table S1) and they did not change transgenerationally following environmental exposures. The DDT, vinclozolin and DDT H3K27me3 induced DHRs are listed in Tables S2, S3 and S4 respectively and the associated genes are listed. For the DDT transgenerational 142 DHRs there were 61 with associated genes. For the vinclozolin transgenerational 99 DHRs there were 44 with associated genes. There were no overlapping genes between the DDT, vinclozolin or DDT H3K27me3 DHR associated genes. Only one gene “U6” overlapped between the DDT and vinclozolin H3 DHR associated genes. The major associated gene categories are presented in Fig. 4d for both the DDT and vinclozolin H3 DHRs and the transcription and signaling functional categories are predominant. The gene pathways associated with the DHRs identified two pathways with three associated genes (metabolic and tight junction pathways) for the DDT H3 DHRs, one pathway with three genes (human papilloma virus infection pathway) for the vinclozolin H3 DHRs, and no pathways with multiple genes for the H3K27me3 DHRs.

## Discussion

A core set of H3 histone retention sites observed in the control lineage male sperm were highly conserved with negligible biological variation. These core retention sites were not altered in the vinclozolin or DDT F3 generation lineage transgenerationally. Previous studies have demonstrated in both mouse and human sperm a set of histone retention sites with specific histone modifications that are associated with important developmental genes^10,18,25^ and infertility^18–20^. A comparison of the identified core set of retention sites with the sites previously described^10,18,22,23^, Table S5, revealed only one overlapping gene “T/BRA”^24^. Due to the inability to alter these core histone retention sites with environmental factors transgenerationally, the proposal is made that these sites have a role in the early zygote following fertilization and allow the paternal genome to contribute to early gene expression, as previously suggested^10,18,22,23,26,27^. As observed with imprinted genes, these epigenetic sites may have evolved to facilitate reproduction and early development in mammals.

The pesticide DDT and fungicide vinclozolin were both found to transgenerationally (i.e. F3 generation) induce the appearance of differential histone retention sites. Preliminary studies indicate no H3 DHRs are observed in the F1 and F2 generations for either vinclozolin or DDT lineage sperm^28^. The transgenerational H3 DHRs developed in addition to the conserved core histone retention sites, which were not altered. However, this observation is dependent on the stringency of DHR and core site identification. This is the first demonstration in mammals for a potential role for histones in environmentally induced epigenetic transgenerational inheritance mechanisms. A previous study showed that genetically altered histone methylation in mice causes generational effects^29^, but this is distinct from the environmentally induced transgenerational phenomenon investigated. The majority of DHRs were found to be intergenic with less than half associated with genes, Tables S2 and S3. These intergenic sites may have a role in the regulation of ncRNAs or influence chromatin structure during early development, so require further investigation. Interestingly, the vinclozolin and DDT induced transgenerational H3 DHRs are exposure specific. Similar observations have been made with DNA methylation and ncRNA transgenerational effects and are thought to have a role in the exposure specific transgenerational impacts observed^1,30^. The ability of two different environmental toxicants to promote transgenerational H3 DHRs provides further support that histone retention sites may have a role in epigenetic transgenerational inheritance.

The histone modification H3K27me3 is a common repressive histone in sperm histones. The analysis of H3K27me3 DHRs in the F3 generation DDT lineage sperm identify a reduced number of DHRs and minimal overlap with the H3 DHRs. The analysis specifically examined differential histone retention between the control and exposure lineage. If no difference in sequencing read depth was observed then no DHR would be identified. Retention sites with the same levels and histone modifications would reduce the number of DHRs detected. The minimal overlap of the H3 DHRs and H3K27me3 DHRs may suggest a lack of alterations in histone modification or similar levels of modification between the control and exposure F3 generation lineages.

The preliminary studies^28^ indicate negligible DHRs in the F1 and F2 generations compared to the high number of DHRs in the F3 generation exposure lineages. The actions of the environmental exposures on the F1 generation fetal exposure and F2 generation direct germline exposure are based on direct exposure mechanisms^1^. In contrast, the F3 generation is transgenerational and based on a reprogramming of the embryonic stem cell population that impacts all subsequent somatic cells and the sperm. Therefore, the direct exposure mechanisms in the F1 and F2 generations do not appear to alter DHRs, but the transgenerational F3 generation mechanism can alter DHRs following ancestral exposures. Therefore, the current study focused on the transgenerational F3 generation sperm DHRs.

The DHR associated genes were found to be predominantly in single gene function categories, including transcription and signaling. No common gene pathways appeared to be influenced by both the vinclozolin and DDT induced transgenerational sperm H3 DHRs. The integration of the core histone associated genes previously identified^10,18,22,23^ and the transgenerational DHR associated genes reported may alter the zygote and early development. These DHRs will likely originate during spermatogenesis in the testis when histones are replaced by protamines to allow compaction of the DNA in the head of the spermatozoa^15^. Epigenetic transgenerational impacts on the testis somatic cells (e.g. Sertoli cells) has been shown to alter germ cell development^31^ so may also be involved in this process. Future studies will need to investigate the origins of the transgenerational DHRs during spermatogenesis and their impacts on the zygote and early embryonic development.

Combined observations demonstrate for the first time in mammals a role for altered histone retention sites in environmentally induced epigenetic transgenerational inheritance. Previous studies have demonstrated transgenerational alterations in DNA methylation^2,5^ and ncRNA^7,8^ in sperm which appear to be involved in epigenetic transgenerational inheritance. The current study suggests an integration of histone modifications and retention with DNA methylation and ncRNA is likely required for a complete understanding of the epigenetic transgenerational inheritance phenomenon. All these epigenetic factors have distinct functions so it is not surprising all appear to be involved in the mechanism. In addition, since epigenetic alterations can promote genetic stability and promote genetic mutations, as previously described^32,33^, the integration with genetic mutations in the future with the transgenerational germline epigenetics is needed. Future research will need to include the histone core and DHRs in the elucidation of epigenetic transgenerational inheritance mechanisms.

## Methods

### Animal studies and breeding

Female and male rats of an outbred Hsd:Sprague Dawley SR®^TM^ (Harlan) at about 70 to 100 days of age were fed ad lib with a standard rat diet and ad lib tap water for drinking. To obtain time-pregnant females, the female rats in proestrus were pair-mated with male rats. The sperm-positive (day 0) rats were monitored for diestrus and body weight. On days 8 through 14 of gestation^34^, the females received daily intraperitoneal injections of vinclozolin (100 mg/kg BW/day), or DDT (25 mg/kg BW/day) or dimethyl sulfoxide (DMSO) in oil (1 µl/kg BW/day vehicle). Vinclozolin and DDT (dichlorodiphenyltrichloroethane) were obtained from Chem Service Inc. (West Chester, PA) and reported to have a purity of 99.5% for vinclozolin and 98.2% for DDT. Vinclozolin and DDT were dissolved and injected in DMSO vehicle as previously described^30^. Treatment lineages are designated “control” or “vinclozolin” or “DDT” lineages. The gestating female rats treated were designated as the F0 generation. The offspring of the F0 generation rats were the F1 generation. Non-littermate females and males aged 70–90 days from F1 generation of control, vinclozolin or DDT were bred to obtain F2 generation offspring. An intercross within the lineages was used to mimic natural populations and avoid altering the phenotype using outcross due to the parent of origin allelic aspects of epigenetic inheritance^1^. The F2 generation rats were similarly bred to obtain the F3 generation offspring. The F1-F3 generation offspring were not themselves treated directly with vinclozolin or DDT, only the F0 generation. For the current study, F3 generation individuals were maintained for 120 days and euthanized for sperm collection. The control, vinclozolin and DDT lineage rats were housed in the same room with the same lighting, food and water as previously described^30,35,36^. All experimental protocols including the rats were pre-approved by the Washington State University Animal Care and Use Committee (IACUC approval # 02568–49). All experiments were performed in accordance with relevant approved guidelines and regulations.

### Epididymal sperm collection

The epididymis was dissected free of connective tissue, a small cut made to the cauda and tissue placed in 3 ml of phosphate buffered saline (PBS) for 20 minutes at room temperature and then kept at 4 °C to immobilize the sperm. The epididymal tissue was minced and then the released sperm centrifuged at 1800x*g* and the pellet resuspended in NIM buffer and stored at −80 °C until processed further. The sample was resuspended and sonicated to destroy any contaminating somatic cells and partially remove the sperm tails. This removed any somatic cell contamination due to the sonication resistance of the sperm heads.

### Histone Chromatin Immunoprecipation (ChIP)

Histone chromatin immunoprecipitation with genomic DNA was performed with a procedure previously described^22^. Individual rat sperm collections were generated and the sperm counts determined for each individual. Equal numbers of sperm were added from each individual for a total of 8 million sperm per pool and 3 pools with different individual rats for each pool for the control, vinclozolin and DDT lineage animals. The control pools contained equal numbers of sperm for each of 5–6 individuals for a total of n = 17 rats, the vinclozolin and DDT pools contained equal numbers of sperm for each of 4 individuals in each pool for a total of n = 12 rats per exposure group. To remove any somatic cell contamination sperm samples from each animal were sonicated 10 seconds using a Sonic Dismembrator Model 300 (Thermo Scientific Fisher, USA) then centrifuged 1800xg for 5 min at 4 °C then resuspended and counted individually on a Neubauer counting chamber (Propper manufacturing Co., Inc., New York, USA) prior to pooling. The sperm pools were reconstituted up to 1 ml with PBS (phosphate buffered saline). To reduce disulfide bonds, 50 µl of 1 M DTT was added to each pool and the pools were then incubated for 2 hours at room temperature under constant rotation. To quench any residual DTT (dithiothreitol, Fisher Scientific, NY USA) in the reaction, 120 µl of fresh 1 M NEM (N-Ethylmaleimide, Thermo Scientific, Rockford, USA) was then added and the samples incubated for 30 min at room temperature under constant rotation. The sperm cells were pelleted at 450 *g* for 5 min at room temperature and the supernatant discarded. Pellets were resuspended in PBS and then spun again at 450 *g* for 5 min at room temperature. The supernatant was discarded.

For the DDT samples we used an enzymatic fragmentation of the chromatin. The sperm cells were then resuspended in “buffer 1” (final concentration: 15 mM Tris-HCl (pH 7.5), 60 mM KCl, 5 mM MgCl2, 0.1 mM EGTA; all filtered through a 0.22-µm filter and stored at room temperature) in a ratio of 2 million sperm cells per 50 µl (as described by Hisano *et al*.^22^). “Complete buffer” was “buffer 1” supplemented with 0.5% tergitol (vol/vol) and 1% DOC (wt/vol) (sodium deoxycholate, Sigma Aldrich 30970). 50 µl of this supplemented buffer was added to each aliquot. The samples were mixed and incubated for 20 min on ice. 10 Kuntz units of MNase (NEB, M0247, Ispwich, MA) were added to each sample and the samples incubated for 5 min at 37 °C. The reaction was stopped by the addition of 2 µl of 0.5 M EDTA.

For the vinclozolin samples we used sonication to fragment the chromatin. Sperm cell DNA was divided into aliquots of 4 µg of DNA. These aliquots were sonicated using the Covaris M220 the following way: 4 µg of genomic DNA was resuspended in 130 µl of complete buffer supplemented with tergitol 0.5% and DOC 1%. Covaris was set to a 10 min “Chromatin shearing” program and the program was run for each tube in the experiment.

After treatment using either MNase or the Covaris sonication, 10 µl of each sample were run on a 1.5% agarose gel to verify fragment size. Aliquoted samples were pooled back together and centrifuged at 12,500 g  for 10 min at room temperature. The supernatant was transferred to a fresh microfuge tube. 65 µl of protease inhibitor cocktail (1 tablet dissolved in 500 µl, 20 × concentrated) (Roche, cat. no. 11 873 580 001) were added in each sample as well as 3 µl of antibody (anti-histone H3 pan-monoclonal antibody, cat no. 05–928, or anti-trimethyl-histone H3 (Lys27) polyclonal antibody, cat no. 07–449, both with broad spectrum species specifically form Millipore Corp, Temecula CA USA). The DNA-antibody mixture was incubated overnight on a rotator at 4 °C. The following day, magnetic beads (ChIP-Grade protein G magnetic beads, Cell Signaling 9006) were pre-washed as follows: the beads were resuspended in the vial, then 30 µl per sample was transferred to a microfuge tube. The same volume of Washing Buffer (at least 1 ml) was added and the bead sample was resuspended. Tube was then placed into a magnetic rack for 1–2 minutes and the supernatant discarded. The tube was removed from the magnetic rack and the beads washed once. The washed beads were resuspended in the same volume of IP buffer as the initial volume of beads. 30 µl of beads were added to each DNA-antibody mixture from the overnight incubation, then incubated for 2 h on a rotator at 4 °C. After the incubation, the bead-antibody-DNA complex was washed three times with IP buffer as follows: the tube was placed into a magnetic rack for 1–2 minutes and the supernatant discarded, then washed with IP buffer 3 times. The washed bead-antibody-DNA solution was then resuspended in 300 µl of digestion buffer (1 M Tris HCI, pH 8.0, 0.5 M EDTA, 10% SDS) and 3 µl proteinase K (20 mg/ml). The sample was incubated for 3 h on a rotator at 56 °C. After incubation the samples were extracted with Phenol-Chloroform-Isoamyalalcohol and precipitated with 2 µl of Glycoblue (20 mg/ml), a one-tenth volume of 3 M sodium acetate and two volumes of ethanol overnight at −20 °C.

The precipitate was centrifuged at 18,000xg for 30 min at 4 °C and the supernatant removed, while not disturbing the pellet. The pellet was washed with 500 µl cold 70% ethanol, then centrifuged again at 18,000xg for 10 min at 4 °C and the supernatant discarded. The tube was spun briefly to collect residual ethanol to bottom of tube and as much liquid as possible was removed with a gel loading tip. Pellet was air-dried at RT until it looked dry (about 5 min) then resuspended in 20 µl H_2_0. DNA concentration was measured in the Qubit (Life Technologies) with the BR dsDNA kit (Molecular Probes Q32853).

### ChIP-Seq Analysis

The ChIP pools were used to create libraries for next generation sequencing (NGS) using the NEBNext® Ultra^TM^ II DNA Library Prep Kit for Illumina® (NEB, Ipswich, MA). The manufacturer protocol was followed. Each pool received a separate index primer. NGS was performed at the WSU Spokane Genomics Core using Ilumina HiSeq. 2500 with a read size of approximately 50 bp PE and approximately 35 million reads per pool. Six libraries were run in one lane.

### Statistics and Bioinformatics

The basic read quality was verified using summaries produced by the FastQC program http://www.bioinformatics.babraham.ac.uk/projects/fastqc/. The raw reads were trimmed and filtered using Trimmomatic^37^. The reads for each ChIP sample were mapped to the Rnor 6.0 rat genome using Bowtie2^38^ with default parameter options. The mapped read files were then converted to sorted BAM files using SAMtools^39^. To identify DHRs, the reference genome was broken into 100 bp windows. The MEDIPS R package^40^ was used to calculate differential coverage between control and exposure sample groups. The edgeR p-value^41^ was used to determine the relative difference between the two groups for each genomic window. Windows with an edgeR p-value less than 10^−7^ (DDT) or 10^−6^ (vinclozolin or DDT–H3K27me3) were considered DHRs. The DHR edges were extended until no genomic window with a p-value less than 0.1 remained within 1000 bp of the DHR.

In addition to the differential analysis, conserved histone sites were identified using an arbitrary read depth threshold of 150 reads. For this analysis, the genome was again broken into 100 bp windows. The windows that met read depth thresholds in all treatment or all control samples were considered conserved histone sites. Neighboring windows were merged into a single site in a similar manner to the DHR. The edges of the conserved site were extended until there were no genomic windows meeting the read depth threshold in all treatment or control samples within 1000 bp of the site edge.

A universal core set of histone retention sites was identified by taking the core sites that overlapped in all six treatment groups (DDT control, DDT treated, vinclozolin control, vinclozolin treated, DDT H3K27me3 treated, and DDT H3K27me3 control). The actual sites that overlap in the different groups may have different lengths and slightly different start and stop positions. For simplicity, the vinclozolin control sites that overlapped with core sites in all other treatment group samples were arbitrary selected to represent the universal core sites.

Histone sites for the five individual rat samples were identified separately from the treatment group core sites. For each individual sample, the histone sites were identified using a read depth threshold of 150. Neighboring genomic windows meeting this threshold were merged if within 1000 bp.

DHRs and conserved sites were annotated using the biomaRt R package^42^ to access the Ensembl database^43^. The genes that overlapped with DHR or conserved site, and those genes that occurred within 10kbp of the DHR or conserved site edge, were then input into the KEGG pathway search^44,45^ to identify associated pathways. The DHR and conserved site associated genes were manually then sorted into functional groups by consulting information provided by the DAVID^46^, Panther^47^, and Uniprot databases incorporated into an internal curated database (www.skinner.wsu.edu under genomic data). All molecular data has been deposited into the public database at NCBI (GEO # GSE106125). The specific scripts used to perform the analysis can be accessed at github.com/skinnerlab and at www.skinner.wsu.edu/genomic-data-and-r-code-files.

## Electronic supplementary material

Supplemental Tables
