# Supplementary material for: Epigenetic Transgenerational Inheritance of Altered Sperm Histone Retention Sites
Source: Sci Rep. 2018 Mar 28;8:5308. doi: 10.1038/s41598-018-23612-y (PMC5871750; doi:10.1038/s41598-018-23612-y)
Supplement: Supplementary file 1 — Supplemental Tables [file 41598_2018_23612_MOESM1_ESM.pdf]

Epigenetic Transgenerational Inheritance of Altered Sperm Histone Retention Sites

Millissia Ben Maamar, Ingrid Sadler-Riggleman, Daniel Beck,
Michael K. Skinner

Supplementary Information

Supplemental Table Legends

Table S1. Core histone retention site list.

Table S2. DDT differential histone retention sites (DHRs) list.

Table S3. Vinclozolin differential histone retention sites (DHRs) list.

Table S4. H3K27me3 DDT differential histone retention sites (DHRs) list.

Table S5. Literature reported histone retention sites list.

Supplemental Table S1
Core Histone Sites

Site Name	Chr #	Start	# Windows	Length (bp)	Gene Annotation	Gene Category
Site1:26599001	1	26599001	1	100		
Site1:148641901	1	148641901	17	2700		
Site1:180644501	1	180644501	17	2200		
Site1:180692001	1	180692001	10	1200		
Site1:180702101	1	180702101	37	4200		
Site1:180718901	1	180718901	9	900		
Site1:180800801	1	180800801	66	9700		
Site1:180852201	1	180852201	7	700		
Site1:180858101	1	180858101	10	1900		
Site1:180861301	1	180861301	2	200		
Site1:180882101	1	180882101	5	500		
Site1:180893101	1	180893101	5	500		
Site1:180896701	1	180896701	12	1800		
Site1:180902601	1	180902601	4	400		
Site1:180911501	1	180911501	20	3000		
Site1:180948901	1	180948901	8	800		
Site1:181185901	1	181185901	7	700		
Site1:181188001	1	181188001	10	1900		
Site1:181286501	1	181286501	6	600		
Site2:197079101	2	197079101	16	2500		
Site3:40701	3	40701	6	1400		
Site3:133994001	3	133994001	31	5800		
Site4:146545701	4	146545701	28	3100		
Site7:22323401	7	22323401	4	400		
Site7:22327201	7	22327201	9	1600		
Site7:100303501	7	100303501	17	1900		
Site10:78149601	10	78149601	1	100	Stxbp4;Cox11	
Site12:1508601	12	1508601	35	4100	5S_rRNA	
Site12:3350301	12	3350301	66	7300		
Site13:33474901	13	33474901	18	2200		
Site13:33502401	13	33502401	31	3100		
Site13:33508401	13	33508401	4	400		
Site13:33591101	13	33591101	25	4000		
Site13:33722901	13	33722901	12	1800		
Site14:28440901	14	28440901	2	200	Adgrl3	
Site17:44391001	17	44391001	5	500		
Site17:44395901	17	44395901	21	4400		
Site18:38412901	18	38412901	49	6200		
Site19:56337701	19	56337701	16	1600	Gas8;5S_rRNA	
Site20:40882001	20	40882001	18	3000		
Site20:40913201	20	40913201	8	800		

Supplemental Table S2
DDT DHRs (p<10-7) List

DHR Name	Chr #	Start	Length (bp)	# SigWin	MinP-Value	CpG #	CpG Density	Associated Gene	Gene Category
DHR1:21653101	1	21653101	800	1	3.40E-10	5	0.62	Enpp3	Signaling
DHR1:52899001	1	52899001	4400	1	9.12E-08	57	1.29	T;T2	Transcription
DHR1:63622201	1	63622201	2800	1	4.98E-08	13	0.46		
DHR1:64723301	1	64723301	800	1	5.03E-08	3	0.37		
DHR1:74435301	1	74435301	900	1	9.18E-08	7	0.77		
DHR1:94639901	1	94639901	3200	1	1.38E-09	22	0.68		
DHR1:96031701	1	96031701	100	1	7.87E-09	0	0		
DHR1:110940801	1	110940801	2400	1	1.54E-08	7	0.29		
DHR1:121353901	1	121353901	1200	1	6.30E-08	5	0.41		
DHR1:158604501	1	158604501	500	1	1.55E-08	2	0.4		
DHR1:191840601	1	191840601	2100	1	5.36E-09	17	0.8	Scnn1b	Transport
DHR1:274030901	1	274030901	16300	1	2.15E-08	482	2.95	Mxi1	Transcription
DHR2:7709401	2	7709401	100	1	6.14E-08	0	0		
DHR2:15915901	2	15915901	200	1	3.26E-08	0	0		
DHR2:17166201	2	17166201	1700	1	2.04E-08	7	0.41		
DHR2:19538201	2	19538201	100	1	6.33E-08	2	2		
DHR2:45199501	2	45199501	600	1	8.21E-08	1	0.16	RGD1561161	Extracellular Matrix
DHR2:55287301	2	55287301	1200	1	4.44E-08	7	0.58		
DHR2:62447001	2	62447001	3800	1	2.70E-09	56	1.47	Pdzd2	Unknown
DHR2:71933501	2	71933501	100	1	9.09E-09	0	0		
DHR2:114565501	2	114565501	100	1	2.27E-10	2	2		
DHR2:120117001	2	120117001	800	1	4.10E-08	6	0.75		
DHR2:132344601	2	132344601	1400	1	9.09E-08	5	0.35	AABR07010330.1; AABR07010330.2	NA
DHR2:143971901	2	143971901	3800	1	9.74E-08	30	0.78	LOC103691556	Development
DHR2:190629101	2	190629101	300	1	2.35E-08	1	0.33	S100vp	Signaling
DHR2:195349901	2	195349901	300	1	4.24E-08	1	0.33		
DHR2:211991601	2	211991601	300	1	6.16E-08	1	0.33		
DHR2:260751601	2	260751601	4400	1	1.81E-08	28	0.63		
DHR3:52348601	3	52348601	2400	1	3.39E-08	29	1.2	Ttc21b	Unknown
DHR3:70634901	3	70634901	3000	1	3.93E-08	21	0.7		
DHR3:100281801	3	100281801	800	1	1.97E-08	5	0.62	Mettl15	Unknown
DHR3:119851001	3	119851001	1500	1	3.13E-09	19	1.26		
DHR3:156431901	3	156431901	4900	1	9.16E-09	72	1.46	AABR07054488.1	NA
DHR3:160151701	3	160151701	1500	1	4.99E-08	7	0.46		
DHR3:161334101	3	161334101	1700	2	4.68E-08	22	1.29	Pcif1	Transcription
DHR3:168069401	3	168069401	9900	1	5.81E-08	207	2.09		
DHR4:6117001	4	6117001	1300	1	6.32E-09	6	0.46	Kmt2c	Transcription
DHR4:28460901	4	28460901	800	1	6.03E-08	5	0.62	Vps50	Unknown
DHR4:29141001	4	29141001	3100	1	8.46E-08	23	0.74		
DHR4:39269301	4	39269301	3600	1	2.40E-08	21	0.58		
DHR4:82386701	4	82386701	4800	1	5.33E-09	47	0.97	AABR07060588.2	NA
DHR4:93787601	4	93787601	1700	1	9.03E-08	3	0.17	AABR07060788.1	NA
DHR4:179456401	4	179456401	4200	1	8.73E-08	78	1.85	Casc1	Unknown
DHR5:7557901	5	7557901	1000	1	3.60E-08	2	0.2		
DHR5:34733901	5	34733901	400	1	2.28E-08	3	0.75	Nkain3	Transport
DHR5:42362001	5	42362001	1500	1	6.51E-08	6	0.4		
DHR5:61265801	5	61265801	2600	1	9.95E-08	26	1		
DHR5:74139401	5	74139401	1400	1	3.36E-08	15	1.07	Epb41l4b	
DHR5:126404301	5	126404301	800	1	7.70E-08	4	0.5	Acot11	Metabolism
DHR5:133465301	5	133465301	600	1	1.54E-09	6	1		
DHR5:151666101	5	151666101	10600	1	6.92E-08	140	1.32	AC118963.2	NA
DHR5:153222501	5	153222501	2000	1	3.27E-08	32	1.6	Rhd;Tmem50a	Transport
DHR5:171384801	5	171384801	19700	1	1.18E-09	383	1.94	Tp73	Transcription
DHR6:490501	6	490501	300	1	9.39E-08	1	0.33		
DHR6:21564501	6	21564501	200	1	3.90E-08	2	1	Ltbp1	Signaling;Extracellular Matrix

DHR6:23770901	6	23770901	500	1	8.47E-08	7	1.4		
DHR6:65733801	6	65733801	8300	1	8.79E-08	29	0.34		
DHR6:72318401	6	72318401	1600	1	4.59E-08	17	1.06		
DHR6:78101601	6	78101601	1300	1	2.53E-08	9	0.69		
DHR6:94845101	6	94845101	4000	1	6.67E-08	70	1.75	Jkamp;Ccdc175	Receptor
DHR7:31228101	7	31228101	300	1	5.96E-08	1	0.33	Anks1b	Receptor
DHR7:36759401	7	36759401	1700	2	1.15E-08	31	1.82		
DHR7:52267801	7	52267801	1200	1	6.30E-08	5	0.41	Nav3	Development
DHR7:101472001	7	101472001	300	1	3.42E-09	1	0.33		
DHR7:108278101	7	108278101	8500	1	9.01E-08	46	0.54		
DHR8:1311701	8	1311701	2700	1	6.65E-08	21	0.77		
DHR8:8987901	8	8987901	5600	1	2.98E-08	40	0.71		
DHR8:11868301	8	11868301	1700	1	3.11E-08	9	0.52		
DHR8:15757901	8	15757901	1100	1	5.86E-08	5	0.45		
DHR8:22587901	8	22587901	3400	1	6.07E-08	36	1.05	Carm1	Epigenetic
DHR8:56663101	8	56663101	1300	1	1.76E-08	15	1.15	Zc3h12c	Transcription
DHR8:104686501	8	104686501	4300	1	6.91E-08	61	1.41		
DHR9:2504001	9	2504001	800	1	5.52E-09	14	1.75		
DHR9:15615301	9	15615301	3700	1	6.92E-08	77	2.08	U6;Guca1a;Guca1b	Signaling
DHR9:44359301	9	44359301	100	1	2.97E-08	0	0	Tsga10	Signaling
DHR10:17789001	10	17789001	1700	2	2.73E-08	18	1.05		
DHR10:19431101	10	19431101	400	1	4.07E-08	13	3.25		
DHR10:30719201	10	30719201	200	1	3.28E-08	1	0.5		
DHR10:33962601	10	33962601	300	1	7.20E-08	2	0.66		
DHR10:74232901	10	74232901	2900	2	3.13E-08	38	1.31	Ypel2	Unknown
DHR10:80208101	10	80208101	2100	1	9.90E-08	5	0.23		
DHR10:86369901	10	86369901	3900	1	3.29E-08	63	1.61	Erbb2;U6	Receptor
DHR10:88076101	10	88076101	2100	1	3.94E-09	23	1.09	Krt14;Rn50_10_0879.7;LOC108352134;Rn50_10_0879.5	Cytoskeleton
DHR10:112214601	10	112214601	1300	1	4.19E-08	23	1.76		
DHR11:713401	11	713401	2000	1	7.57E-08	16	0.8	Epha3	Receptor
DHR11:2657201	11	2657201	300	1	6.93E-08	4	1.33	Pou1f1	Transcription
DHR11:16751201	11	16751201	1500	1	1.36E-08	8	0.53		
DHR11:24413001	11	24413001	2500	1	9.23E-08	31	1.24	App	Signaling
DHR11:75080801	11	75080801	3400	1	4.41E-08	58	1.7	Atp13a5	Transport
DHR11:81865301	11	81865301	500	1	9.81E-09	1	0.2		
DHR12:3207201	12	3207201	100	1	6.21E-08	1	1	AABR07035008.3;AABR07035008.1;AABR07035008.2	NA
DHR12:12345301	12	12345301	1700	1	3.95E-08	24	1.41		
DHR12:30870401	12	30870401	5100	2	6.60E-11	59	1.15		
DHR12:31592301	12	31592301	2700	1	4.80E-09	48	1.77	Rimbp2	Binding Protein
DHR12:41347101	12	41347101	9400	1	2.01E-08	132	1.4	Oas2	Transcription
DHR12:44655701	12	44655701	5100	2	3.43E-08	68	1.33	Ksr2	Signaling
DHR12:49132601	12	49132601	800	1	1.77E-08	2	0.25		
DHR12:49999601	12	49999601	1200	1	6.00E-09	7	0.58		
DHR13:35160901	13	35160901	7100	1	5.50E-08	85	1.19		
DHR13:98634901	13	98634901	5800	1	6.00E-08	101	1.74	Itpkb	Signaling
DHR13:105938801	13	105938801	700	1	1.63E-08	7	1		
DHR13:107153901	13	107153901	800	1	6.92E-09	6	0.75	Ush2a	Extracellular Matrix
DHR13:108923601	13	108923601	3900	1	6.90E-08	75	1.92		
DHR14:12569201	14	12569201	400	1	4.47E-09	2	0.5		
DHR14:27374201	14	27374201	1300	1	9.37E-08	12	0.92		
DHR14:45699901	14	45699901	1900	1	8.42E-10	65	3.42	Tbc1d1	Signaling
DHR14:48414001	14	48414001	4300	1	5.82E-08	20	0.46		
DHR14:66240201	14	66240201	3000	1	8.46E-08	15	0.5		
DHR14:73199901	14	73199901	1800	1	4.98E-08	11	0.61		

DHR14:83665301	14	83665301	1000	1	3.89E-10	5	0.5	Rnf185	Transcription
DHR14:110472901	14	110472901	400	1	9.05E-08	2	0.5		
DHR15:16826901	15	16826901	1100	1	2.68E-08	10	0.9		
DHR15:23225501	15	23225501	3900	1	4.18E-08	17	0.43		
DHR15:29085801	15	29085801	900	1	6.26E-09	3	0.33	AABR07017599.1	NA
DHR15:66487401	15	66487401	100	1	1.38E-08	0	0	SNORA17	NA
DHR15:71154301	15	71154301	500	1	8.16E-08	2	0.4		
DHR15:74103601	15	74103601	900	1	1.16E-08	4	0.44		
DHR15:97162101	15	97162101	2000	1	5.00E-08	5	0.25		
DHR16:1520801	16	1520801	2000	1	2.94E-09	30	1.5		
DHR16:2434801	16	2434801	200	1	4.16E-08	1	0.5	Dnah12	Cytoskeleton
DHR16:12322101	16	12322101	100	1	6.44E-09	1	1		
DHR16:54006501	16	54006501	1900	1	1.63E-08	27	1.42	Asah1	
DHR16:68733201	16	68733201	5900	1	8.91E-08	70	1.18		
DHR16:77797201	16	77797201	1700	1	6.01E-08	13	0.76		
DHR16:80659701	16	80659701	6400	1	1.70E-09	208	3.25	Erich1	Unknown
DHR16:81037201	16	81037201	1100	1	6.28E-08	3	0.27	Tmco3	Transport
DHR16:85488501	16	85488501	1500	1	5.85E-08	8	0.53		
DHR17:24965901	17	24965901	1900	1	5.45E-08	25	1.31		
DHR17:30107501	17	30107501	1000	1	9.41E-08	13	1.3		
DHR17:61918901	17	61918901	1200	1	2.56E-08	6	0.5		
DHR17:88432001	17	88432001	900	1	1.79E-08	15	1.66	Gpr158	Receptor
DHR18:25095501	18	25095501	1700	1	7.26E-08	20	1.17		
DHR18:44107301	18	44107301	1500	1	8.99E-09	4	0.26	LOC103694210	NA
DHR18:46655901	18	46655901	1500	1	2.55E-09	10	0.66		
DHR18:47777701	18	47777701	1700	1	9.71E-08	19	1.11	Sncaip	Development
DHR19:3174201	19	3174201	2800	1	5.36E-08	17	0.6		
DHR19:52418801	19	52418801	1900	1	7.40E-08	32	1.68	Tldc1	Unknown
DHR19:52551601	19	52551601	3300	1	6.30E-08	41	1.24		
DHR19:60051001	19	60051001	700	1	9.12E-08	8	1.142	Pard3	Cell Junction
DHR20:13453401	20	13453401	1000	1	8.89E-08	16	1.6	Slc5a4	Transport
DHR20:45575901	20	45575901	500	1	9.12E-08	6	1.2	Cdk19	Cell Cycle
DHR20:48972401	20	48972401	2200	1	2.62E-08	69	3.13	Aim1	Development

Supplemental Table S3
Vinclozolin DHRs (p<10-6) List

DHR Name	Chr #	Start	Length (bp)	# SigWin	MinP-Value	CpG #	CpG Density	Associated Gene	Gene Category
DHR1:20114501	1	20114501	300	1	1.61E-07	14	4.66	LOC100360791	
DHR1:26098101	1	26098101	1900	1	6.47E-08	12	0.63		
DHR1:38831601	1	38831601	2400	1	6.46E-07	21	0.87	AABR07001207.1; AABR07001210.1	NA
DHR1:56022601	1	56022601	16600	1	1.81E-10	128	0.77		
DHR1:91780301	1	91780301	200	1	3.50E-07	1	0.5	Tdrd12	Transcription
DHR1:130661501	1	130661501	600	1	1.76E-07	4	0.66	AABR07004221.2; AABR07004221.1	NA
DHR1:148050901	1	148050901	1900	1	6.98E-07	6	0.31	LOC100361547	NA
DHR1:148469701	1	148469701	800	1	6.79E-07	11	1.37		
DHR1:162732901	1	162732901	200	1	7.13E-07	0	0	AC133383.1	NA
DHR1:172681501	1	172681501	1100	1	1.55E-07	15	1.36		
DHR1:179450901	1	179450901	8200	1	9.62E-08	75	0.91		
DHR1:182254101	1	182254101	2000	1	3.86E-07	22	1.1		
DHR1:197921801	1	197921801	1500	1	9.78E-07	15	1	Atxn2l	Development;Unknown
DHR1:258374601	1	258374601	1100	1	6.45E-08	4	0.36		
DHR1:267314801	1	267314801	1200	1	4.14E-07	58	4.83	Stn1	Signaling
DHR2:7491201	2	7491201	1200	1	1.46E-07	7	0.58		
DHR2:8302401	2	8302401	2600	1	4.55E-07	33	1.26		
DHR2:46298001	2	46298001	1400	1	3.01E-07	9	0.64		
DHR2:158865101	2	158865101	8400	1	5.76E-07	65	0.77		
DHR2:159989201	2	159989201	3900	1	1.88E-07	38	0.97		
DHR2:160707201	2	160707201	1900	1	8.34E-10	6	0.31		
DHR2:161417501	2	161417501	2800	1	4.30E-07	18	0.64		
DHR2:206393801	2	206393801	1900	1	5.79E-07	16	0.84	Ptpn22	Signaling
DHR3:40557601	3	40557601	600	1	1.85E-07	1	0.16	Galnt13	Unknown
DHR3:64849201	3	64849201	4800	2	4.63E-07	45	0.93	AABR07052589.1	NA
DHR3:96269401	3	96269401	1100	1	3.21E-08	5	0.45		
DHR3:96406901	3	96406901	1100	1	7.67E-07	3	0.27		
DHR3:99311301	3	99311301	4300	1	4.01E-07	41	0.95		
DHR3:106526601	3	106526601	400	1	9.96E-07	2	0.5		
DHR3:134798701	3	134798701	2700	1	6.49E-07	9	0.33		
DHR3:169128301	3	169128301	3900	1	9.29E-07	25	0.64		
DHR4:43876701	4	43876701	5400	1	3.87E-07	37	0.68		
DHR4:70613001	4	70613001	800	1	7.28E-07	2	0.25	AC142181.1;Prss3	NA;Proteolysis
DHR4:72255501	4	72255501	400	1	9.28E-07	13	3.25		
DHR4:84869401	4	84869401	200	1	1.93E-07	2	1	Mturn	
DHR4:131323001	4	131323001	100	1	8.91E-08	1	1		
DHR4:143416201	4	143416201	800	1	7.12E-07	3	0.37	U6	Translation
DHR5:4747501	5	4747501	300	1	5.10E-07	1	0.33	Rbpj	Transcription
DHR5:111459001	5	111459001	500	1	5.86E-07	7	1.4		
DHR6:32404601	6	32404601	400	1	1.89E-09	10	2.5	AABR07063508.1	NA
DHR6:40324101	6	40324101	800	1	6.14E-07	2	0.25		
DHR6:50579901	6	50579901	600	1	8.22E-08	8	1.33	Lamb1	Extracellular Matrix
DHR6:70047001	6	70047001	1300	1	4.55E-07	7	0.53		
DHR6:139316601	6	139316601	400	1	1.89E-07	0	0		
DHR7:9633901	7	9633901	1100	1	1.36E-08	3	0.27	Olr1072	Receptor
DHR7:13470601	7	13470601	2000	1	6.53E-07	19	0.95	Olr1077	Receptor
DHR7:21201301	7	21201301	800	1	7.83E-07	3	0.37	AABR07056200.1	NA
DHR7:21932501	7	21932501	9200	1	2.17E-07	41	0.44		
DHR7:27041001	7	27041001	300	1	6.50E-07	6	2		
DHR7:73133501	7	73133501	700	1	1.81E-07	1	0.14	Matn2	Extracellular Matrix
DHR7:139312601	7	139312601	1000	1	3.03E-07	29	2.9	Hdac7	Epigenetic
DHR7:142476401	7	142476401	1300	1	6.08E-07	24	1.84		
DHR8:10561601	8	10561601	500	1	5.26E-07	2	0.4		
DHR8:64174701	8	64174701	300	1	3.23E-07	0	0	Arih1	Protease

DHR8:106250001	8	106250001	500	1	4.23E-07	6	1.2		
DHR9:11690901	9	11690901	200	1	6.17E-07	5	2.5		
DHR9:45398701	9	45398701	200	1	3.58E-08	0	0		
DHR9:89873301	9	89873301	1700	1	8.11E-07	12	0.7		
DHR9:111560601	9	111560601	1500	1	9.78E-07	20	1.33		
DHR10:52882501	10	52882501	1400	1	5.85E-08	14	1	Shisa6	Development
DHR11:3960701	11	3960701	1700	1	9.78E-07	11	0.64		
DHR11:23267901	11	23267901	300	1	7.17E-07	3	1	U4	Translation
DHR12:34170901	12	34170901	2400	1	8.11E-07	20	0.83		
DHR13:18134301	13	18134301	1800	1	1.14E-07	5	0.27		
DHR13:37396601	13	37396601	100	1	9.78E-07	0	0	Ccdc93	Unknown
DHR13:53512701	13	53512701	400	1	2.04E-07	8	2	Zfp281	Transcription
DHR13:69789901	13	69789901	700	1	7.97E-07	4	0.57	Tsen15	Translation
DHR13:82823801	13	82823801	1700	1	6.11E-07	25	1.47		
DHR13:88115001	13	88115001	2100	1	2.54E-07	20	0.95	AABR07021730.1	NA
DHR13:89436001	13	89436001	11900	1	1.03E-07	475	3.99	Fcgr2b	Immune
DHR13:91203701	13	91203701	400	1	4.21E-08	26	6.5	LOC100911825; LOC108348047	Receptor
DHR14:21520801	14	21520801	100	1	8.24E-09	0	0	Smr3b	Development
DHR14:77890801	14	77890801	3300	1	6.48E-08	48	1.45	Stk32b	Signaling
DHR14:80322501	14	80322501	5000	1	2.85E-07	68	1.36	Acox3	Metabolism
DHR14:100736101	14	100736101	1100	1	9.14E-07	25	2.27		
DHR15:18327301	15	18327301	1600	1	6.61E-07	13	0.81	Fam3d	Unknown
DHR15:21107801	15	21107801	1100	1	3.65E-08	9	0.81	Rn50_15_0251.1	NA
DHR15:62619901	15	62619901	2100	1	8.92E-07	29	1.38		
DHR15:66768001	15	66768001	2000	1	6.15E-07	14	0.7		
DHR16:7904201	16	7904201	1100	1	1.93E-07	7	0.63	Ankrd28	Unknown
DHR16:11145501	16	11145501	1000	1	2.03E-08	23	2.3		
DHR16:62074501	16	62074501	300	1	8.59E-07	10	3.33	Rbpms	Transcription
DHR17:46967501	17	46967501	200	1	8.07E-07	2	1		
DHR17:53391501	17	53391501	2200	1	6.01E-07	6	0.27	Hecw1	Protease
DHR17:89407701	17	89407701	12000	1	7.28E-07	112	0.93		
DHR18:9297101	18	9297101	500	1	1.07E-07	5	1		
DHR18:23356401	18	23356401	100	1	4.07E-08	0	0		
DHR18:30445201	18	30445201	3300	1	7.35E-07	23	0.69	Pcdhb8	Cytoskeleton
DHR18:40199401	18	40199401	300	1	4.40E-07	3	1	Pggt1b	Transport
DHR18:61681401	18	61681401	200	1	8.57E-07	2	1	Cplx4	Golgi
DHR19:28125101	19	28125101	10900	1	2.51E-07	92	0.84	AABR07043407.1	NA
DHR19:59823501	19	59823501	200	1	3.53E-07	2	1		
DHR20:26763401	20	26763401	200	1	8.11E-07	3	1.5	Herc4	Protease
DHR20:27460201	20	27460201	4500	1	1.34E-07	172	3.82		
DHR20:27471801	20	27471801	3300	2	9.67E-09	143	4.33		
DHR20:36595601	20	36595601	400	1	5.63E-07	2	0.5		
DHR20:54854301	20	54854301	300	1	3.96E-07	5	1.66		
DHRX:103932101	X	103932101	200	1	9.13E-07	2	1		
DHRX:150631501	X	150631501	300	1	5.19E-07	1	0.33		

Supplemental Table S4
DDT H3K27me3 DHRs List

DHR Name	Chr #	Start	Length (bp)	# SigWin	MinP-Value	CpG #	CpG Density	Associated Gene	Gene Category
DHR2:6073501	2	6073501	600	1	7.34E-07	3	0.5		
DHR2:240590901	2	240590901	700	1	2.77E-07	14	2	SNORA26	Binding Protein
DHR5:6372701	5	6372701	2000	19	7.98E-33	33	1.65	AABR07046778.1	NA
DHR5:167331301	5	167331301	100	1	7.88E-07	17	17	Rere	Epigenetic
DHR8:181201	8	181201	1100	10	1.21E-15	24	2.18		
DHR10:75365201	10	75365201	1200	2	1.85E-08	115	9.58	Vezf1	Transcription
DHR15:56665201	15	56665201	200	1	1.03E-07	7	3.5	Htr2a	Receptor
DHR19:8431801	19	8431801	300	1	6.60E-07	7	2.33		
DHR20:1729401	20	1729401	500	1	4.51E-07	3	0.6	Olr1734	Receptor
DHR20:19484001	20	19484001	1800	9	4.56E-19	22	1.22	Fam13c	Unknown
DHRMT:1	MT	1	16300	124	1.43E-26	303	1.85	Mt-co	Metabolism

Supplemental Table S5
Literature Histone Retention Sites List

Author	Histones	Genes	techniques
Hisano M, Erkek S, Dessus-Babus S, Ramos L, Stadler MB, Peters AH. (2013) Genome-wide chromatin analysis in mature mouse and human spermatozoa. Nat Protoc. 8(12):2449-70.	"nucleosome enrichment"	Pold3_1 Pold3_2 Rest_1 Rest_2 Nanog Intergenic Gata6 Sfrs6 Sall4 Foxd3	qPCR and ChIP
	H3K27me3	Kdm5a Sfrs6 Prmt5 Cdx2 Bmp4 Sox2	qPCR and ChIP
Hammoud SS, Nix DA, Zhang H, Purwar J, Carrell DT, Cairns BR. (2009) Distinctive chromatin in human sperm packages genes for embryo development. Nature. 460(7254):473-8.	"nucleosome enrichment"	Sox2 Nanog Oct4	ChIP
Brykczynska U, Hisano M, Erkek S, Ramos L, Oakeley EJ, Roloff TC, Beisel C, Schübeler D, Stadler MB, Antoine, Peters AHFM (2010) Repressive and active histone methylation mark distinct promoters in human and mouse spermatozoa. Nature Structural & Molecular Biology 17, 679–687.	H3K4me2	**PROMOTERS** TSH2B Pgk2 Actl7a Tnp1 Prm1 Brdt Sfrs6 Jarid1a Rps3 Taf8 Dicer1 Prmt5 Rest Pml Tcl1a Myc Gdf3 Stra8 Sall4 Tbx3 Prdm1 Lif gata6 Foxd3 Zic3 T Sox2 Bmp4 Cdx2 Evx1 Hoxa1 Hoxa4 Hoxa9 Hoxc6 Hoxc10 Hoxd8 Nanog Oct4	ChIP
	H3K27me3	**PROMOTERS** TSH2B Pgk2 Actl7a Tnp1 Prm1 Brdt Sfrs6 Jarid1a Rps3 Taf8 Dicer1 Prmt5 Rest Pml Tcl1a Myc Gdf3 Stra8 Sall4 Tbx3 Prdm1 Lif gata6 Foxd3 Zic3 T Sox2 Bmp4 Cdx2 Evx1 Hoxa1 Hoxa4 Hoxa9 Hoxc6 Hoxc10 Hoxd8 Nanog Oct4	ChIP and qPCR
Vieweg M, Dvorakova-Hortova K, Dudkova B, Waliszewski P, Otte M, Oels B, Hajimohammad A, Turley H, Schorsch M, Schuppe HC, Weidner W, Steger K, Paradowska-Dogan A. (2015) Methylation analysis of histone H4K12ac-associated promoters in sperm of healthy donors and subfertile patients. Clin Epigenetics. 19;7:31.	H4K12ac	**PROMOTERS** AFF4, EP300, LRP5, RUVBL1, USP9X, NCOA6, NSD1, and POU2F1	pyrosequencing (human sperm healthy donors and subfertile patients)
